# LEDGINs inhibit late stage HIV-1 replication by modulating integrase multimerization in the virions

**DOI:** 10.1186/1742-4690-10-57

**Published:** 2013-05-30

**Authors:** Belete Ayele Desimmie, Rik Schrijvers, Jonas Demeulemeester, Doortje Borrenberghs, Caroline Weydert, Wannes Thys, Sofie Vets, Barbara Van Remoortel, Johan Hofkens, Jan De Rijck, Jelle Hendrix, Norbert Bannert, Rik Gijsbers, Frauke Christ, Zeger Debyser

**Affiliations:** 1 Department of Pharmaceutical and Pharmacological Sciences, Laboratory for Molecular Virology and Gene Therapy, KU Leuven, Kapucijnenvoer 33, Leuven, Flanders, 3000, Belgium; 2 Laboratory for Photochemistry and Spectroscopy, KU Leuven, Celestijnenlaan 200F, Heverlee, Flanders, 3001, Belgium; 3 Robert Koch Institute, Centre for HIV and Retrovirology, Nordufer 20, Berlin, 13353, Germany

**Keywords:** Antivirals, HIV replication, Integrase, Integrase multimerization, LEDGINs

## Abstract

**Background:**

LEDGINs are novel allosteric HIV integrase (IN) inhibitors that target the lens epithelium-derived growth factor (LEDGF)/p75 binding pocket of IN. They block HIV-1 integration by abrogating the interaction between LEDGF/p75 and IN as well as by allosterically inhibiting the catalytic activity of IN.

**Results:**

Here we demonstrate that LEDGINs reduce the replication capacity of HIV particles produced in their presence. We systematically studied the molecular basis of this late effect of LEDGINs and demonstrate that HIV virions produced in their presence display a severe replication defect. Both the late effect and the previously described, early effect on integration contribute to LEDGIN antiviral activity as shown by time-of-addition, qPCR and infectivity assays. The late effect phenotype requires binding of LEDGINs to integrase without influencing proteolytic cleavage or production of viral particles. LEDGINs augment IN multimerization during virion assembly or in the released viral particles and severely hamper the infectivity of progeny virions. About 70% of the particles produced in LEDGIN-treated cells do not form a core or display aberrant empty cores with a mislocalized electron-dense ribonucleoprotein. The LEDGIN-treated virus displays defective reverse transcription and nuclear import steps in the target cells. The LEDGIN effect is possibly exerted at the level of the Pol precursor polyprotein.

**Conclusion:**

Our results suggest that LEDGINs modulate IN multimerization in progeny virions and impair the formation of regular cores during the maturation step, resulting in a decreased infectivity of the viral particles in the target cells. LEDGINs thus profile as unique antivirals with combined early (integration) and late (IN assembly) effects on the HIV replication cycle.

## Background

HIV-1 integrase (IN) is responsible for the insertion of viral reverse transcribed double-stranded genomic DNA into host chromatin. The integration process proceeds through two canonical reactions called 3’ processing and strand transfer [1]. The first reaction requires at least a dimer of IN on each viral DNA end, while a dimer of dimers binding both ends is required for the second [2,3]. It is generally believed that a dynamic equilibrium between different oligomeric states of IN in time and space is essential for the completion of the HIV life cycle [2,3]. A shift in the multimerization equilibrium of IN may perturb its catalytic activities and structural functions in the preintegration complexes (PICs) resulting in defective integration [4]. Integration of lentiviruses including HIV is dictated by the specific interaction between IN and the cellular cofactor lens epithelium-derived growth factor (LEDGF/p75) that acts as a molecular tether linking IN to the chromatin [5-10].

Integrase is an attractive target for drug development. All HIV IN inhibitors currently in the clinic belong to the class of IN strand transfer inhibitors (INSTIs) that target the active site of IN bound to processed viral DNA. This class includes raltegravir, elvitegravir and dolutegravir (a second-generation INSTI in phase-III clinical trials) [11], all potent antivirals with high safety profiles. However, resistance readily emerges in patients against these inhibitors. Therefore, development of next-generation IN inhibitors preferably targeting alternative sites of the enzyme is a major priority in the field of antiviral research.

In search of such inhibitors, we recently discovered a novel class of small molecule IN inhibitors targeting the LEDGF/p75 binding pocket located at the dimer interface of the IN catalytic core domain (CCD) [12]. The compounds within this class are hence referred to as LEDGINs (LEDGF/p75-IN interaction site) [13]. Due to the allosteric nature of LEDGINs, recently it has been proposed to change the name to ALLINIs (allosteric integrase inhibitors) [14]. ALLINIs though refers to all inhibitors which do not directly interfere with the catalytic site of integrase. Thus it is a generalized name of different classes of integrase inhibitors with distinct mechanisms of actions as reviewed by Neamati et al.[15], and does not refer to the specific and novel mechanism of action of LEDGINs. LEDGINs inhibit replication of all HIV-1 clades tested at submicromolar concentration and show no cross-resistance with INSTIs [16]. Apart from disrupting the LEDGF/p75-IN interaction, LEDGINs and their analogs allosterically inhibit the catalytic activities of IN by perturbing its multimerization state [12,14,16,17]. Furthermore, we recently reported that LEDGINs seem to affect the replication capacity of progeny virions [16]. The objectives of the current study were to investigate the molecular basis of the antiviral activity of LEDGINs in the late stage of HIV-1 replication and pinpoint the defects in the progeny virions and during the subsequent viral life cycles in target cells. We demonstrate that LEDGINs are able to engage IN in the context of the Pol polyprotein and modulate its multimerization. LEDGINs augment intravirion IN multimerization and prevent the formation of regular cores in a significant proportion of viral particles thereby strongly impairing the replication capacity without affecting proteolytic cleavage or genomic RNA (gRNA) packaging.

## Results

### Replication capacity of progeny virus grown in the presence of LEDGINs is reduced

The replication capacity of HIV-1 particles produced by chronically infected HuT78 cells in the presence of LEDGINs seems to be impaired [16]. Before determining the molecular basis of the late effect of LEDGINs, we corroborated this observation by examining the replication capacity of virus produced in the presence of LEDGINs. HuT78 cells chronically infected with HIV-1_IIIB_ (referred herein as HuT78_IIIB_ cells) were grown in the presence of different concentrations of LEDGINs (CX04328; EC_50_ = 2.35 μM or CX05045; EC_50_ = 0.76 μM) [12]. As controls, we included antivirals that inhibit HIV reverse transcription (AZT; EC_50_ = 0.02 μM), integration (raltegravir; EC_50_ = 0.006 μM) and proteolytic maturation (ritonavir; EC_50_ = 0.06 μM). The 50% effective concentrations (EC_50_) were determined in an MTT/MT-4 assay and used to calculate the concentration of compounds added in the various assays.

**Figure 1:**
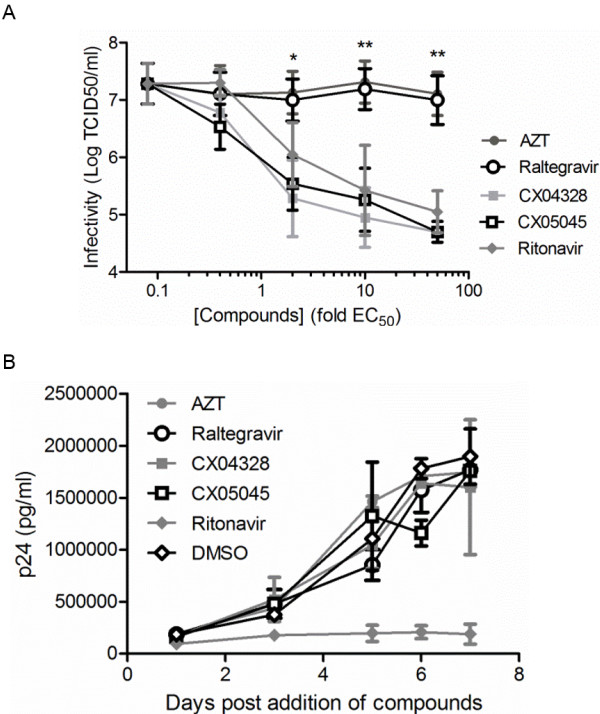
**LEDGINs impair replication capacity of HIV**-**1 produced by HuT78**_**IIIB **_**cells. **HIV-1_IIIB _was produced by persistently infected HuT78_IIIB_ cells in the presence of different concentrations of AZT, raltegravir, CX04328, CX05045, ritonavir or DMSO. (**A**) Replication capacity of these viruses was assayed in MT-4 cells and data represent virus titer as determined by 50% tissue culture infectivity dose (logTCID_50_/ml) (mean values ± standard deviations; n = 3). (**B**) Kinetics of p24 production over successive days was measured for cells treated with 10-fold EC_50 _of the inhibitors (n = 2). *p<0.05, **p < 0.01; 2-way ANOVA.

The replication capacity of HIV-1_IIIB_ produced by HuT78_IIIB_ in the presence of increasing concentrations of AZT or raltegravir was evaluated in MT-4 cells. Replication of progeny virus was not affected compared to DMSO-treated cells with an average infectivity of 7.3±0.62 log TCID_50_/ml (Figure 1A). In contrast, viruses produced in the presence of ritonavir or LEDGINs displayed a concentration dependent impairment of productive infection. At concentrations of 50-fold their EC_50_ values, ritonavir and LEDGIN reduced the cytopathic effect of viruses more than 100-fold in comparison with viruses produced in the presence of DMSO, AZT or raltegravir (2-way ANOVA, p<0.01) (Figure 1A). Concomitantly, we monitored the kinetics of virus production by HuT78_IIIB_ cells in the presence of compounds at concentrations equal to 10-fold the EC_50_ value (Figure 1B). Except for ritonavir, none of the tested inhibitors affected the accumulation of p24 in the supernatant as monitored by p24 ELISA.

### LEDGINs inhibit multiple steps in HIV replication

**Figure 2:**
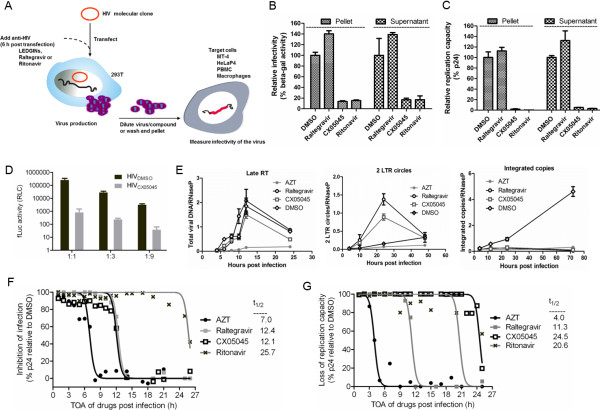
**Systematic evaluation of the multiple effects of LEDGINs on HIV**-**1 replication.** (**A**) Schematic representation of virus production and infectivity assays to determine the late effect of LEDGINs. (**B**, **C**) Detailed analysis of the late effect of LEDGINs on the replication of NL4.3 (**B**) in HeLaP4 cells as determined by measuring beta-galactosidase activity 24 hpi (mean values ± standard deviations; n = 2), (**C**) in MT-4 cells by measuring p24 72 hpi (mean values ± standard deviations; n = 2). (**D**) Infectivity of HIV-1-IN-eGFP/VSV.G pseudovirus [27] generated in the presence CX05045 (HIV_CX05045_) or DMSO (HIV_DMSO_) as measured by Firefly luciferase (fLuc) activity (mean values ± standard deviations of triplicate measurements for three dilutions of inocula normalized for p24 antigen are shown). (**E**) qPCR analysis for the kinetics of late RT product, 2-LTR circles and integrated copies in MT-4 cells are shown (mean values ± standard deviations of triplicate measurements are plotted). (**F**, **G**) Time-of-addition (TOA) analysis in MT-4 cells for (**F**) inhibition of single round replication determined at 31 hpi or (**G**) loss of replication capacity of the progeny virions was determined 4 dpi by p24 ELISA in the supernatants. Time (t_1/2,_ h) of compound addition yielding 50% inhibition is indicated for each compound. As expected, AZT loses activity at 4–7 hpi and raltegravir at 11–12 hpi coinciding with RT and integration steps, respectively. Ritonavir affects p24 production and virus infectivity at around 20–26 hpi. CX05045 profiles as (**F**) an integration inhibitor (t_1/2 _= 12.2 h) when assaying single round infection_, _(**G**) whereas the late effect is evidenced only when measuring replication capacity of the progeny virions released in its presence (t_1/2_ = 24.5 h). Data represent normalized inhibition to the DMSO control (set at 100%).

LEDGINs are known to target IN at the LEDGF/p75-IN interaction interface and block integration [12]. Because LEDGINs also curtail the replication capacity of virus produced from chronically infected HuT78 cells (Figure 1A), we set up a series of assays to unambiguously dissect their effects during the different stages of HIV replication. First, we produced virus by transfection of 293T cells in the presence of CX05045 (5 μM), raltegravir (0.03 μM), ritonavir (0.3 μM) or DMSO (no-inhibitor) and investigated infectivity of the progeny virions in different cells (Figure 2A). To eliminate the possibility that compound is carried over in the supernatant together with the virus, we also used viruses that were extensively washed and pelleted by ultracentrifugation. We then examined the replication capacity of the viruses in HeLaP4 and MT-4 cells by measuring beta-galactosidase activity and p24 protein in the supernatants at 24 and 72 h post infection (hpi), respectively. Unlike raltegravir and irrespective of the extensive washing, ritonavir and CX05045 profoundly impaired virus replication when added during production (Figure 2B, C), ruling out that the effect is caused by the carry-over of compound in the supernatant. To further corroborate the late effect of LEDGINs on infectivity of HIV-1, we produced single round VSV.G pseudotyped HIV pseudovirus in the presence or absence of CX05045 and measured the firefly luciferase (fLuc) activity in MT-4 cells. Addition of CX05045 during production resulted in lower fLuc activity (e.g. >300-fold for the 1:1 dilution) compared to the DMSO-treated virus (Figure 2D).

We then examined the replication cycle of HIV in time using qPCR analysis of viral DNA species [18] and time-of-addition (TOA) [19]. Consistent with our previous report on the mode of action of LEDGINs in the early stage of HIV replication [12], CX05045 blocks HIV-1 integration without affecting the upstream replication events (Figure 2E). While only AZT inhibited RT activity, both CX05045 and raltegravir significantly blocked integration resulting in an accumulation of 2-long terminal repeat (2-LTR) circles at 24 hpi (Figure 2E), a hallmark of IN inhibitors [12,20,30].

Next, we designed and carried out a TOA experiment in MT-4 cells in which the antivirals were added every hour post infection and the supernatants were harvested 31 hpi, the average duration of a single HIV replication cycle in laboratory-adapted T cells [21,22]. Theoretically, addition of a drug after the completion of the step targeted will result in a lack of inhibition and hence p24 protein will accumulate in the supernatant. As such, the targeted step by CX05045 or the control inhibitors (AZT, raltegravir and ritonavir) was monitored by quantifying p24 protein in the supernatants harvested from the TOA experiment (Figure 2F, Additional file 1: Table S1). The average time delay post infection (t_0_) when addition of the compound retained 50% inhibition of HIV-1 replication (t_1/2_) was calculated [23]. Accordingly, we found t_1/2_ of ~ 7.0, 12.4, 12.1 and 25.7 hpi for AZT, raltegravir, CX05045 and ritonavir, respectively (Figure 2F, Additional file 1: Table S1). These correspond to RT (AZT), integration (raltegravir and the early effect of CX05045) and proteolytic maturation steps (ritonavir). Subsequently, to pinpoint the late effect of LEDGINs, we used the supernatants harvested from the TOA experiment and evaluated the replication capacity of the progeny virions. To do this, we infected new MT-4 cells with the supernatants and quantified p24 protein in the supernatants 4 days post infection (dpi) (Figure 2G, Additional file 1: Table S1). As expected, cells incubated with supernatants harvested from cells treated with AZT (beyond 4 hpi) or raltegravir (beyond 11 hpi) in the TOA experiment displayed comparable productive infection as the control virus (DMSO-treated) infected cells, coinciding with their targets i.e. RT and integration, respectively (Figure 2G). On the other hand, viruses produced in the presence of ritonavir added as late as 21 hpi in the TOA experiment were less infectious, corresponding to the proteolytic maturation block (Figure 2G). Remarkably, when monitoring replication capacity of viruses produced in the presence of CX05045, we found that the viruses displayed impaired replication capacity when CX05045 was added as late as 24 hpi (Figure 2G). These results clearly establish that LEDGINs affect both integration and late stages of HIV replication. To assess the relative contribution of both effects, we determined EC_50_ values for the early and the late effect using a beta-galactosidase assay (Table 1). CX05045 blocks HIV integration and virion infectivity in HeLaP4 cells with EC_50_ values of 4.45±2.34 μM and 1.46±0.01 μM, respectively, indicating that both effects contribute to the overall inhibition of multiple round HIV replication (1.14±0.32 μM) (Table 1).

**Table 1:** **Stage**-**specific antiviral activity of LEDGINs**

	**EC**_**50 **_**(μM)**		
	**Multiple round**^**a**^	**Early stage**^**b**^	**Late stage**^**b**^
CX05045	1.14±0.32	4.45±2.34	1.46±0.01
Raltegravir	0.006±0023	0.0031±0.0013	> 3
Ritonavir	0.05±0.0027	>3	0.04±0.006

^a ^Effective concentration required to reduce HIV-1 induced cytopathic effect by 50% in MT-4 cells as determined by an MTT/MT-4 assay. ^b^ Effective concentration required to reduce Tat-driven beta-galactosidase expression by 50% in HeLaP4 cells at 24 hpi. Mean values ± standard deviations from at least two independent experiments are shown.

### LEDGINs do not affect virion gRNA packaging or proteolytic cleavage but interfere with the assembly of regular mature cores

**Figure 3:**
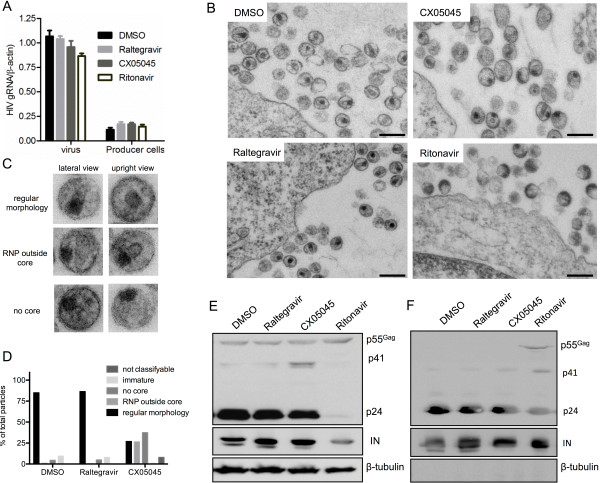
**Characterization of the replication defect induced by LEDGIN treatment. **(**A**) Viral gRNA and total RNA extract of the respective producer cells as quantified by RT-qPCR. Mean values ± standard deviations of triplicate measurements from two experiments are shown. (**B**, **C**) Morphology of viral particles produced from HuT78_IIIB_ cells grown in the presence of DMSO, raltegravir, CX05045 or ritonavir. (**B**) Representative TEM overview images are presented. (**C**) TEM images exemplifying regular and aberrant particle morphology in the LEDGIN treated sample. (**D**) The particles were classified and quantified according to their morphology in thin section EM. The percentage was calculated relative to the total number of particles analyzed (200–400 particles per condition). The ritonavir-treated sample contained > 90% immature particles (not shown). (**E**, **F**) Western blot analysis of viral proteins in HuT78_IIIB_ producer cells or the corresponding cell-free viruses produced in the presence of the indicated compounds. β-tubulin was used as loading control. Scale bars represent 500 nm.

We next explored potential mechanisms underlying the late effect of LEDGINs. We first examined the impact of CX05045, raltegravir or ritonavir on the efficiency of gRNA packaging by RT-qPCR analysis and on the morphology of HIV-1 particles by transmission electron microscopy (TEM) (Figure 3A-C). None of the inhibitors interfered with gRNA packaging (1-way ANOVA, p>0.05) (Figure 3A).

TEM analysis of the morphology of viral particles at or near the plasma membrane clearly demonstrated that ritonavir affected virus maturation rendering almost all of the particles released to be immature (Figure 3B) [24]. Interestingly, while no morphological differences to the DMSO control have been noticed in the raltegravir treated sample, particles with a mislocalized electron-dense ribonucleoprotein (RNP) and particles lacking a core structure were frequently observed in the CX05045 sample (Figure 3B and C). A quantitative analysis classifying 200–300 visualized particles per sample revealed that about 26% of the virions display an aberrant empty core with an external RNP frequently attached to the virus membrane and rarely to the empty core. The empty core was usually thinner than regular cores and often bar shaped. In 37.5% of the particles no core was visible at all and the electron dense RNP complex was attached to the virus membrane (Figure 3C). A regular core with the RNP generally localized at the broader site of the conical core was present in only 27% of the CX05045 treated particles but in 85% of the DMSO control and 86.5% of the raltegravir sample (Figure 3D).

To investigate the viral precursor polyprotein processing pattern, Western blot analysis was performed on samples from virus producer HuT78_IIIB_ cells as well as on virus lysate produced in the presence of DMSO, raltegravir, CX05045 or ritonavir. In contrast to the expected effect of ritonavir on viral protein processing, we observed no significant effect on Gag polyprotein processing in the producer cells (Figure 3D) and on virus released in the supernatants (Figure 3E), correlating with p24 (Figure 1B) and morphology analysis (Figure 3B, C). Taken together, these data indicate that LEDGINs impair HIV infectivity (i.e. a late effect) through a mechanism distinct from proteolytic cleavage or gRNA packaging. LEDGINs clearly affect the formation of a regular mature core containing the RNP.

### The late effect of LEDGINs requires a direct interaction with HIV-1 integrase

**Figure 4:**
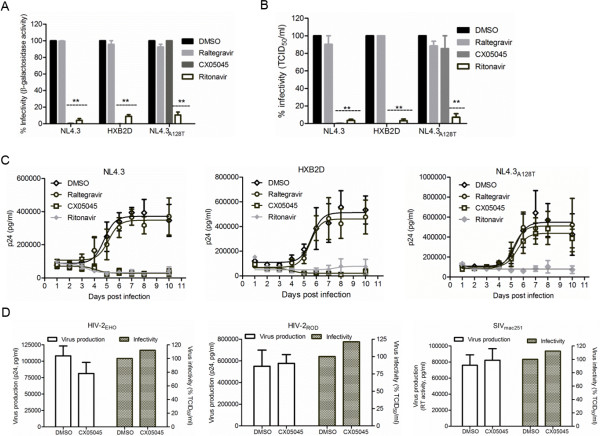
**Reduction of virion replication capacity by LEDGINs is integrase dependent.** Detailed analysis of the late effect of LEDGINs on the replication capacity of different HIV-1 strains (NL4.3, LEDGIN-resistant NL4.3_A128T _or HXB2D): (**A**) in HeLaP4 cells as determined by measuring beta-galactosidase activity 24 hpi (n = 2), (**B**) in MT-4 cells by calculating the TCID_50_/ml 5 dpi (n = 4), and (**C**) in PBMC by monitoring p24 level in the supernatant (n = 2–4). (**D**) Effect of CX05045 on HIV-2 and SIV_mac251 _production and infectivity. The replication capacity of the viruses was determined by scoring CPE in MT-4 cells on day 5 postinfection as determined by relative to TCID_50_/ml (n = 2). All data represent mean values ± standard deviation. **p < 0.001; 2-way ANOVA.

LEDGINs, the result of structure-based drug design targeting IN, were shown to bind to the LEDGF/p75 binding pocket in IN by crystallography [12]. If the impairment of HIV replication capacity by LEDGINs is mediated by a direct interaction with IN at the LEDGF/p75 binding pocket, productive infection of the LEDGIN-resistant strain NL4.3_A128T_, [12] should not be hampered by addition of LEDGINs during virus production. In line with this, we produced NL4.3_A128T_ and different wild type (WT) strains (NL4.3, HXB2D and YU-2) in the presence of CX05045, raltegravir, ritonavir or DMSO, and monitored virus replication in HeLaP4 cells, MT-4 cells, peripheral blood mononuclear cells (PBMC) or monocyte derived macrophages (MDM) as shown in Figure 2A. We compared the replication of WT and NL4.3_A128T_ viruses in HeLaP4 (Figure 4A), MT-4 cells (Figure 4B) and PBMC (Figure 4C). The replication of NL4.3 and HXB2D produced in the presence of CX05045 was reduced 200- and 1,750-fold in HeLaP4 and 200- and 2,600-fold in MT-4 cells, respectively, compared to DMSO or raltegravir pretreatment (2-way ANOVA, p<0.001) (Figure 4A, B). In stark contrast, NL4.3_A128T_ replication was unaffected (2-way ANOVA; p>0.05) (Figure 4A, B). As expected, all HIV-1 strains produced in the presence of ritonavir displayed a statistically significant 10- to 30-fold drop in viral replication in HeLaP4 and MT-4 cells (2-way ANOVA, p<0.001) (Figure 4A,B).

Of note, in activated human PBMC isolates, X4-tropic HIV-1 (NL4.3, HXB2D) hardly replicated when produced in the presence of either CX05045 or ritonavir compared to DMSO or raltegravir (Figure 4C). Replication of NL4.3_A128T_ in PBMC was only impaired when produced in the presence of ritonavir but not CX05045 (Figure 4C). To further confirm the specificity of the late effect of LEDGINs, we also tested HIV-2 (HIV-2_EHO_ and HIV-2_ROD_) and SIV_mac251_ (Figure 4D). These viruses have a methionine residue at position 128 of their INs, resulting in a natural resistance to LEDGINs [12]. Consistent with our hypothesis, CX05045 did not affect the replication capacity of HIV-2 or SIV_mac251_ (Figure 4D). We also observed severely hampered productive infections of X4- (NL4.3 and HXB2D) and R5-tropic (YU-2) viruses in MT-4 cells and MDM, respectively, when quantifying the p24 level in the supernatants over successive days (Additional file 1: Figure S1). Collectively, these results suggest that the late antiviral effect of LEDGINs is mediated through a direct interaction with the LEDGF/p75 binding pocket on IN without affecting proteolytic cleavage or gRNA packaging (Additional file 1: Figure 2SA,B).

### Virions generated in the presence of LEDGINs display replication defects in reverse transcription and nuclear import

**Figure 5:**
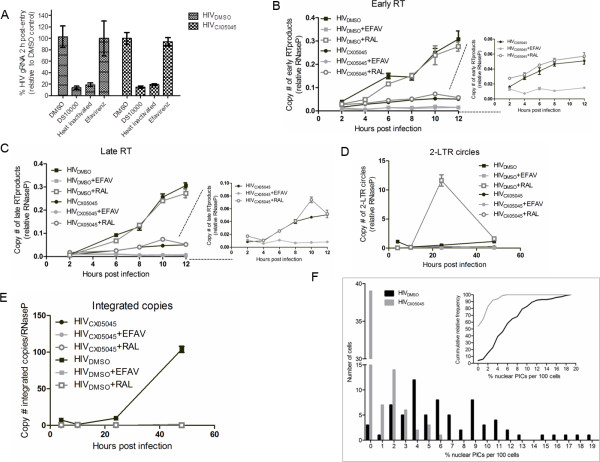
**Systematic evaluation of the multiple effects of LEDGINs on HIV**-**1 replication.** (**A**-**E**) (RT)-qPCR analyses for gRNA and viral DNA species evaluating early replication events (entry to integration) are shown. (**A**) HIV_CX05045_ enters cells as efficient as the control (HIV_DMSO_) virus as measured by RT-qPCR analysis of gRNA isolated 2 h after synchronized infection of MT-4 cells. As expected, addition of DS10000, but not efavirenz, inhibits entry of both HIV_DMSO_ and HIV_CX05045_. Heat-inactivated virus preparation was used as negative control. Mean values ± standard deviation from three independent experiments are shown. (**B**, **C**) qPCR analysis for early and late RT product kinetics in MT-4 cells infected with HIV_DMSO _or HIV_CX05045 _in the presence or absence of efavirenz (EFAV) or raltegravir (RAL) added during infection as RT and integration inhibitor controls. There is a five-fold less RT products in cells infected with HIV_CX05045 _compared to HIV_DMSO _and efavirenz blocked the RT activity of both viruses. (**D**, **E**) 2-LTR circle and integrated copies kinetics are shown. HIV_DMSO _has normal nuclear import as well as integration which are evidenced by the accumulation of 2-LTR circles in cells treated with raltegravir. In stark contrast to HIV_DMSO_, the PICs of HIV_CX05045_ did not translocate into the nucleus as shown by background integrants. This result was further supported by the lack of the accumulation of 2-LTR circles when cells were treated with raltegravir (HIV_CX05045_+RAL). Mean values ± standard deviations for triplicate measurements. (**F**) Analysis of HIV preintegration complex (PIC) nuclear import. Percentage of nuclear versus total PICs in cells infected with HIV_CX05045 _(*gray bars*, n = 72) or HIV_DMSO _(*black bars*, n = 71) is shown. More than 50% of cells infected with HIV_CX05045_ did not contain any fluorescently labeled PIC in the nucleus. In contrast, 2-7% of fluorescently labeled PICs were detected in the nucleus of more than 50% of cells infected with HIV_DMSO_. In the inset, the cumulative probability of nuclear PICs is plotted for HIV_CX05045 _(*gray*) and HIV_DMSO_ (*black*). The empirical distribution function was computed using the Kolmogorov-Smirnov test, p < 0.0001).

To pinpoint the replication defect(s) of virus produced in the presence of CX05045 during the subsequent replication cycle, we produced HIV-1_IIIB_ in the presence of CX05045 (HIV_CX05045_) or DMSO (HIV_DMSO_) and infected MT-4 cells after normalizing for p24 protein. Next, (RT)-qPCR analyses were carried out on cellular extracts obtained at different time points after infection to assess the effect on virus entry and early replication events. HIV_CX05045_ entered cells as efficiently as HIV_DMSO_ in a synchronized infection as determined by quantification of gRNA by RT-qPCR analysis at 2 hpi (Figure 5A). As expected, heat inactivation of the virus or addition of the entry inhibitor DS10000, but not the RT inhibitor efavirenz, resulted in reduced gRNA copy number (Figure 5A).

We next examined the RT step by profiling viral DNA synthesis kinetics using qPCR analysis. Compared to HIV_DMSO,_ we observed a five-fold drop in the levels of both early and late reverse transcripts in from HIV_CX05045_ infected cells extracts at 12 hpi (Figure 5B, C). Efavirenz blocked reverse transcription of both viruses as evidenced by background level of both early and late RT products (Figure 5B, C), demonstrating that HIV_CX05045_ carries functional RT. Of note, CX05045 inhibits RT neither in vitro (data not shown) nor in vivo (Figure 2E) [12]. Compared to HIV_DMSO_ infected cells, background levels of 2-LTR circles (Figure 5D) and integrated copies (Figure 5E) were evidenced in cells infected with HIV_CX05045_, suggesting that the virus displays additional defects at the nuclear import step. As expected, the integration block incurred by raltegravir during infection was accompanied by an increase in 2-LTR circles in cells infected with HIV_DMSO_ (HIV_DMSO_+RAL; more than 20-fold higher circles compared to HIV_DMSO_ at 24 hpi). However, we observed a background level of 2-LTR circles in HIV_CX05045_ infected cells, which remained identical even after raltegravir treatment (only 1.4-fold increase compared to untreated HIV_CX05045_ at 24 hpi) (Figure 5D), suggesting that there is little or no viral cDNA translocated into the nucleus.

The reduced number of 2-LTR circles raised the question whether HIV_CX05045_ is also defective for nuclear import of the PIC, an event believed to be at least partially dependent on the dynamic interaction between IN carried in the PIC and karyopherins [25,26]. To address this issue, we performed a nuclear PIC import assay using fluorescently labeled HIV-1 particles [27]. We produced VSV.G pseudotyped particles, carrying fluorescently labeled IN (IN-eGFP) through Vpr-mediated transincorporation, in the presence of CX05045 (HIV_CX05045_) or DMSO (HIV_DMSO_). HeLaP4 cells were infected with either HIV_CX05045_ or HIV_DMSO_ after normalizing for p24 antigen. The catalytically inactive IN_D64E_ encoded by the proviral construct was successfully transcomplemented by the Vpr-fused IN-eGFP as determined by fLuc activity at 48 hpi (Figure 2D). In two independent experiments, the cellular distribution of the PICs was analyzed in HeLaP4 cells at 7 hpi and the number of nuclear and total PICs was quantified by confocal microscopy. For HIV_DMSO_ and HIV_CX05045_ infected samples, 71 and 72 cells were analyzed, respectively. We detected 7.1 ± 0.83% and 0.45 ± 0.13% of fluorescently labeled PICs in the nucleus for HIV_DMSO_ or HIV_CX05045_, respectively (Figures 5F, Additional file 1: Figure S3). In addition, an analysis of the cumulative distribution probability revealed a statistically significant difference between HIV_DMSO_ and HIV_CX05045_ (p<0.0001; non-parametric two-tailed Kolmogorov-Smirnov test) (Figure 5F, inset). Taken together, these data demonstrate that LEDGIN-induced loss in infectivity is based on defects in reverse transcription and nuclear import.

### LEDGINs modulate IN multimerization in the nascent viral particles

**Figure 6:**
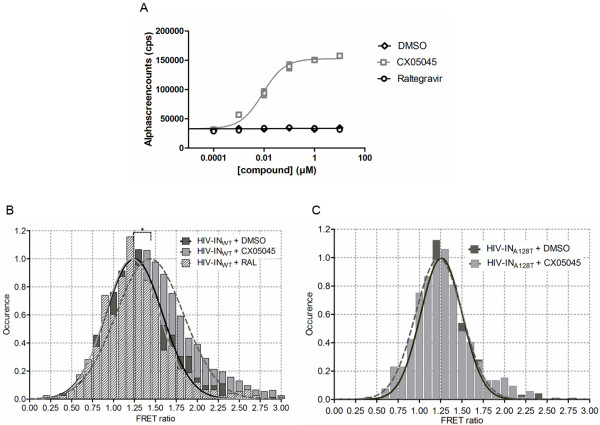
**LEDGINs enhance multimerization of HIV integrase. **(**A**) Direct and specific interaction of LEDGINs with HIV-1 Pol polyprotein. (**A**) Titration of CX05045 or raltegravir against a constant background of 33 nM His-MBP-sPol-PR_D25N_ and 33 nM GST-sPol-PR_D25N_. DMSO (vehicle) included as no-inhibitor control. Only CX05045 was able to enhance the Pol-Pol multimerization with an EC_50_ of 8.7 nM (95% CI 5.7-13.4). Data represent mean values ± standard deviations of duplicate measurements in two independent experiments. (**B**, **C**) FRET analysis of IN multimerization in the virions. Histograms were plotted from pooled data of two independent experiments for (**B**) wild type HIV-1 (HIV-IN_WT_) and (**C**) LEDGIN-resistant (HIV-IN_A128T_) virions. (**B**) FRET ratios for populations of fluorescent HIV-IN_WT _virions produced in the presence of DMSO (*dark gray bar*), CX05045 (*light gray bar*) or RAL (*hatched bar*) along with the respective normal distribution fits for DMSO (*dark line*), RAL (*light gray line*) and CX05045 (*broken line*) are shown. The calculated mean and 95% CI of the FRET ratios for HIV-IN_WT _are 1.25 (95% CI 1.23-1.28) (DMSO), 1.22 (95% CI 1.19-1.25) (RAL) and 1.43 (95% CI 1.42-1.45) (CX05045). (**C**) FRET ratios for populations of fluorescent HIV-IN_A128T_ virions produced in the presence of DMSO (*dark gray bar*) or CX05045 (*light gray bar*) along with the respective normal distribution fits for DMSO (*dark gray line*) and for CX05045 (*broken line*) are shown. The calculated mean and 95% CI of the FRET ratios for HIV-IN_A128T _when produced in the presence or absence of CX05045 are 1.23 (95% CI 1.21-1.25) and 1.26 (95% CI 1.24-1.27), respectively. A mean FRET ratio that equals unity means no FRET. *p <0.05; Student *t* test.

During progeny virion assembly and budding, IN is part of the precursor Gag-Pol polyprotein. As LEDGINs are able to enhance IN multimerization in vitro [16], we hypothesized that the multimerization of the precursor Pol polyprotein may similarly be influenced by LEDGINs through their specific interaction with IN and thereby impacting the generation of infectious particles. Using an AlphaScreen protein-protein interaction (PPI) assay, we examined the effect of CX05045 on Pol polyprotein multimerization using recombinant Glutathione S-Transferase tagged (GST)-Pol and His-Maltose-Binding Protein (MBP)-tagged Pol polyproteins both containing a catalytically dead protease (PR_D25N_). We observed that CX05045 strongly enhanced Pol multimerization in a concentration-dependent manner with an EC_50_ of 8.7 nM (95% CI 5.7-13.4), whereas the raltegravir and DMSO controls had no effect on Pol multimerization (Figure 6A). These results indicate that LEDGINs are able to interact with IN as part of the precursor Pol polyprotein and modulate its multimerization.

Next we investigated whether LEDGINs can perturb the dynamics of IN multimers in nascent virions. To address this issue, we set up an assay based on single-molecule Förster Resonance Energy Transfer (FRET) (Borrenberghs et al., unpublished results). Fluorescently labeled chimeric HIV particles (herein referred to as HIV-IN_WT_) were produced using Vpr-mediated trans-incorporation of IN-mTFP1 (FRET donor) and IN-mVenus (FRET acceptor) in the presence of DMSO, CX05045 or raltegravir. The fluorescence intensity of IN donor per virion was quantified before and after photobleaching of IN acceptor by a combination of total internal reflection and quantitative super-resolution localization microscopy. As shown in Figure 6B the FRET ratio, which is a measure of the amount of dequenching of the IN-donor after photobleaching of IN-acceptor, is significantly larger than unity when virions were produced in the presence of DMSO with a mean of 1.25 (95% CI 1.23-1.28), proving that IN multimerization in the virion can be measured with this assay. HIV-IN_WT_ virions produced in the presence of raltegravir showed a similar mean FRET ratio of 1.22 (95% CI 1.19-1.25). When virions were produced in the presence of CX05045, the mean FRET ratio increased to 1.43 (95% CI 1.42-1.45) (*p*<0.05), strongly suggesting that LEDGINs enhance IN multimerization in the virion, consistent with previous in vitro data with recombinant IN [14,16,17,28]. The specificity of this effect of LEDGINs was further corroborated by examining the impact of CX05045 on the multimerization of LEDGIN-resistant HIV-IN_A128T_ in the virions produced the same way as the HIV-IN_WT_ particles. HIV-IN_A128T_ virus showed comparable FRET ratio when produced in the presence or absence of CX05045 with mean FRET ratio of 1.23 (95% CI 1.21-1.24) and 1.26 (95% CI 1.25-1.27), respectively (Figure 6C). In conclusion, addition of LEDGINs during virus production enhances IN multimerization, which results in HIV-1 particles with severe maturation defects and hampered infectivity.

## Discussion

LEDGINs, potent allosteric HIV integration inhibitors, are designed as small molecule PPI inhibitors targeting the interaction between LEDGF/p75 and IN [12]. By occupying the LEDGF/p75 binding pocket on the IN dimer interface, LEDGINs enhance IN multimerization and therefore allostericly interfere with its catalytic activities [14,16,17]. In addition we recently reported the late stage antiviral effect of LEDGINs [16]. However, detailed analysis and elucidation of the mechanistic basis for the antiviral effect of LEDGINs in the late stage of HIV-1 replication is essential to guide the further development of combination therapy including this class of inhibitors and will provide insight into the possible role of the LEDGF/p75-IN interaction in the late stage of HIV replication [29].

In a series of experiments we unambiguously demonstrate that LEDGINs impair the infectivity of progeny virions through their direct interaction with IN during the late stage of HIV replication. The infectivity of viruses produced in the presence of LEDGINs is significantly reduced without affecting proteolyic cleavage or gRNA packaging (Figures 1, 2, 3A). Instead, the severely impaired infectivity is attributed to enhanced IN multimerization in progeny virions (Figure 6), resulting in aberrant core maturation (Figure 3). This leads to abortive reverse transcription and nuclear import steps in the next replication round (Figures 4–5). In other words, while LEDGINs block HIV integration, a hallmark shared with other integrase inhibitors [12,20,30], they intrinsically also exert an at least equipotent antiviral activity (Table 1) during the late stage of HIV replication, which establishes LEDGINs as a unique class of antiretrovirals.

LEDGINs clearly enhance IN oligomerization in vitro [12,14,16,17] and in the viral particle (Figure 6B). The question remains whether the interaction between IN and LEDGINs may already take place in the configuration of the Pol precursor. This would require Pol dimerization since the LEDGF/p75 pocket is only present in the IN dimer. We tried to answer this question by performing a Pol dimerization assay in the AlphaScreen format (Figure 6A). LEDGINs clearly enhanced Pol multimerization at nanomolar concentrations. These data suggest that LEDGINs potently induce Pol dimerization as a result of enhanced IN dimerization and imply that low amounts of LEDGINs may in fact be specifically bound to IN in the viral particle.

Initial characterization of the antiviral activity of LEDGINs demonstrated that they block HIV-1 integration by disrupting the LEDGF/p75-IN interaction and by allosteric inhibition of the integrase catalytic activity [14,16,17]. The data presented here do not only confirm inhibition at the integration step, but extend the mechanism of action of LEDGINs to late stages of HIV replication. Modulation of the equilibrium of IN multimers in the virions by LEDGINs is likely to perturb their dynamics in the viral particle with deleterious consequences for core formation during the maturation process. Consistent with results obtained with two other LEDGINs recently presented by Yant and co-workers [31] CX05045 treatment of the producer cells prevented the assembly of regular electron-dense cores in two thirds of the virions and almost half of those displayed an abnormal core with an external ribonucleoprotein (RNP) usually attached to the viral membrane. These irregular particles and the virions that manage to form a morphologically normal core are able to enter a target cell, but are defective for RT and nuclear import. The reported impact of IN alterations on the morphology of the viral core is not without precedence. The phenotype of empty cores with misplaced RNP was previously observed with IN mutants [32]. It will be interesting to unravel the underlying mechanism leading to a similar phenotype in these mutants and in viruses produced in the presence of LEDGINs.

With respect to modulating IN multimerization Meehan, et al., previously reported on dominant interference by green fluorescent protein-tagged IN binding domain of LEDGF/p75 (GFP-IBD) when overexpressed in stringent LEDGF/p75 knock-down cells. A durable inhibition of HIV replication was attributed to premature or improper IN multimerization and inhibition of integration [33]. We propose that the dominant interference effect of the IBD of LEDGF/p75 in fact extends to the late stage of HIV replication as well and could contribute to the near complete inhibition of spreading HIV infections [33]. As such, it is possible that the interaction between IN and LEDGF/p75 may be required in the late stage of HIV replication, which is further supported by the late effect of LEDGF/p75-binding cyclic peptides identified as specific LEDGF/p75-IN interaction inhibitors [29]. Therefore, the late effect of LEDGINs may additionally involve a block in the interaction between LEDGF/p75-IN in the late stage of HIV replication, and expose LEDGF/p75-stripped IN to proteasomal degradation in infected cells [33-35]. These mutually non-exclusive mechanisms await further experimentation.

Our findings hold translational relevance. Recently, the superior antiviral activity of non-nucleoside reverse transcriptase inhibitors (NNRTIs) and especially protease inhibitors (PIs) has been explained by steep dose–response curves and cooperativity [36,37]. Positive cooperativity (slope >1) results in a high instantaneous inhibitory potential (IIP) of compounds in a single round HIV-1 infection assay [37]. A Hill coefficient of 3.9 was reported for CX04328 (LEDGIN 6) [14]. Authors attributed this value to the multimodal mechanism of LEDGINs during integration. We likewise observed a high cooperativity for the late antiviral effect of LEDGINs (data not shown) and therefore the late effect of LEDGINs likely contributes to the high cooperativity observed [14]. Of note, some NNRTIs have been implicated to increase dimerization of Gag-Pol polyproteins in virus producer cells and prematurely activate PR affecting protein cleavage and virion maturation [38]; this mechanism possibly contributes to the steep dose–response curve of NNRTIs. Unlike other antiretroviral drugs, viruses generated in the presence of PIs display defective RT in subsequent infections [39,40], explaining their high cooperativity. In any case LEDGINs are unique in targeting IN molecules during both early and late steps of HIV replication explaining the high cooperativity of this novel class of antivirals and increasing their clinical potential [36,37]. Interestingly, unlike NNRTIs [38], LEDGINs do not seem to increase premature PR activation as no effect on proteolytic cleavage and virus production was observed. Although LEDGINs are strong enhancers of Pol multimerization (Figure 6A), we did not observe an increase in premature PR activation and processing of precursor viral polyproteins in the producer cells (Figure 3).

## Conclusions

Our results explain the molecular basis of the late effect of LEDGINs, representing a unique antiviral mechanism. Although inhibition of integration has received most attention, the late effect of LEDGINs can complement the effect on integration and shows high cooperativity in reducing productive infection. Given the complexities of HIV replication, the application of this novel class of inhibitors will permit to unravel previously unidentified but important pathways to further our understanding on the biology of HIV. Moreover, the multi-step antiviral mode of action of LEDGINs is a clinically relevant observation that increases the therapeutic potential of this class of antivirals to complement the current therapeutic arsenals.

## Methods

### Ethics statement

The human peripheral blood mononuclear cells were isolated from anonymous healthy blood donors’ Buffy coats obtained from the University hospitals Gasthuisberg Leuven Blood Bank, as approved by the ethical committee of the University Hospitals Gasthuisberg Leuven.

### Reagents

Antiviral compounds. LEDGINs (CX04328, CX05045) were synthesized by Centre for Drug Design and Development (CD3), KU Leuven R&D, Leuven, Belgium. DS10000, AZT, efavirenz, raltegravir and ritonavir were obtained from AIDS Research and Reference Reagent Program, Division of AIDS, NIH).

Antibodies. Anti-β-tubulin (mouse,T-4026, Sigma-Aldrich, St Louis, MO), anti-HIV-1 CA^p24^ (mouse, #24-2, AIDS Research and Reference Reagent Program, Division of AIDS, NIAID, NIH), anti-HIV-1 IN (mouse, IN-2 (ab66645), Abcam plc, Cambridge Science Park, Cambridge, UK were used.

### Cell culture

293T and HeLaP4 cells were maintained in Dulbecco’s modified Eagle medium (GIBCO BRL, Merelbeke, Belgium) supplemented with 8% fetal calf serum (FCS; Sigma-Aldrich, Bornem, Belgium) and 50 μg/ml gentamicin (GIBCO BRL). MT-4, HuT78, and HuT78_IIIB_ cells were grown in RPMI 1640 (GIBCO BRL) supplemented with 12% FCS and 50 μg/ml gentamicin. Human peripheral blood mononuclear cells (PBMC) were purified from fresh buffy coats of anonymous voluntary donors using Lymphoprep (Axis-Shield PoC AS, Oslo, Norway) following the manufacturer’s protocol. Subsequently, PBMC were maintained and stimulated in RPMI 1640 supplemented with 15% FCS, 20 U/ml IL-2 and 10 μg/ml PHA for three days before use in the infectivity assay. To prepare human monocyte derived macrophages (MDM), PBMC were purified as described above. Subsequently monocytes were isolated from PBMC through depletion of non-monocytes by MACS Cell Separation Columns (MACS; Miltenyi Biotec, Leiden, the Netherlands). 2x10^6^ monocytes/well of a 6-well plate were seeded in RPMI (supplemented with 10% FCS, 100 ng/ml of Macrophage Colony Stimulating Factor (MCSF) and 50 μg/ml gentamicin). Differentiation was done for 7 days. All cell lines were grown in a humidified atmosphere with 5% CO_2_ at 37°C.

### Virus strains

All HIV-1 (NL4.3, IIIB, NL4.3_A128T_, and HXB2D), HIV-2 (ROD, EHO) and SIVmac251 strains were described before [12,41]. Virus titer was determined by microscopically scoring of HIV- induced cytopathic effect (CPE) in MT-4 cells.

### Virus production from chronically infected HuT78 cells

Chronically HIV-1_IIIB_ infected HuT78 cells (here called HuT78_IIIB_) were generated by incubating cells with HIV-1_IIIB_ at a MOI of 0.001-0.01 for at least three weeks; virus release in the supernatant was monitored by p24 quantification using p24 ELISA (Innogenetics, Ghent, Belgium). For virus production, HuT78_IIIB_ cells were washed 3 times with PBS and incubated with different concentrations of AZT, raltegravir, CX04328, CX05045, ritonavir or DMSO. 24–36 h post addition of the compounds, cells were washed again twice with PBS and incubated in fresh medium supplemented with the respective compound for 6 more days and cell-free supernatants were harvested and kept at −80°C until use.

### Virus production by transfection

Production of different HIV-1 molecular clones (NL4.3, NL4.3_A128T,_ HXB2D or YU-2) was carried out by transfecting 293T cells as described before [42]. Briefly, 5.5×10^6^ cells were plated with 5% FSC supplemented DMEM and transfected the following day with 20 μg of plasmid per cell culture dish in OptiMEM without serum. The transfection mix was added directly on the cells drop by drop and 6 h posttransfection, the transfection medium was replaced with 50 μg/ml gentamicin supplemented OptiMEM with or without raltegravir (0.03 μM), CX05045 (5 μM) or ritonavir (0.3 μM). 72 h posttransfection, cell-free supernatants were harvested and filtered through 0.22 μm filters (Millipore). In selected cases virus preparations were washed twice times with PBS using Vivaspin (50 kDa Cut-off, Millipore) while centrifuging at 3,000×g. The third wash was done while pelleting by ultracentrifugation (27,500 rpm, 2 h, SW28 rotor, Beckman Coulter, Fullerton, CA). The pellets were resuspended in PBS and the virus aliquots were stored at −80°C until use.

### Analysis of viral genomic RNA packaging

Virus was produced by transfection as described above using serum-free medium (OptiMEM). 48 h post transfection, supernatants were harvested, filtered through 0.22 μm filters, pelleted by ultracentrifugation (31,000 rpm, 45 minutes, Ti70 rotor, Beckman Coulter, Fullerton, CA), and resuspended in 100 μl PBS. Producer cells (3x10^6^ cells per condition) were also collected, washed, and pelleted. Prior to RNA extraction, non-infected 293T cells (1x10^6^) were added to each virus sample to control for the efficiency of RNA extraction, for cDNA synthesis and for qPCR quantification normalization. Total RNA was extracted both from the producer cells and virus preparations to quantify viral genomic RNA (gRNA) using Total RNA Mini Kit (BioRad, Nazareth, Belgium) following the manufacturer’s recommendations. 5 μg of total RNA was used for cDNA synthesis using the High capacity cDNA reverse transcription kit (Applied Biosystems, Foster City, CA). As a negative control, an equivalent amount of RNA from uninfected cells was used. Genomic RNA was quantified using qPCR with primers Gag_1_ (5'-ATCAAGCAGCCATGCAAATGTT-3') and Gag_2_ (5’-CTGAAGGGTACTAGTAGTTCCTGCTATGTC-3’) and the TaqMan Gag probe (5'-(FAM)-GACCATCAATGAGGAAGCTGCAGAATGGGA-(TAMRA)-3'). To determine the relative amount of gRNA viral transcripts, cDNA corresponding to human β-actin was amplified and used as an internal control for normalization. All samples were run in triplicate for 3 minutes at 95°C followed by 40 cycles of 10 seconds at 95°C and 30 seconds at 55°C. Data were analyzed with iQ5 Optical System Software (BioRad).

### HIV replication assays

Equal amounts of viruses normalized for p24 antigen were used to determine infectivity in different cells (MT-4, HeLaP4, PBMC and MDM) with or without washing. To determine the 50% tissue culture infective dose (TCID_50_), a serial 5-fold dilution of virus was done in triplicate on MT-4 cells (96-well format, 3×10^4^ cells per well). 5 dpi, wells containing infected cells were identified by the presence of cytopathic effect (CPE), and the TCID_50_ was calculated according to the Spearman-Karber protocol. Data are presented as relative infectivity compared to controls (DMSO-treated).

To determine replication capacity we used viruses with or without washing three times. The viruses were pelleted by ultracentrifugation (27,5000 rpm for 2 h using an SW28 rotor, Beckman Coulter). All infection experiments were performed after normalization for p24 protein. 2×10^5^ HeLaP4 cells were seeded per well in 24-well plates and infections were carried out the next day using 2–6 μg of p24 equivalent virus. Cells were lysed, and HIV Tat-driven beta-galactosidase activity (24 hpi) and HIV fLuc activity (72 hpi) were quantified using the β-Gal reporter gene assay (Roche diagnostics, Mannheim, Germany) and Steady-Glo Luciferase assay (Promega), respectively, according to the manufacturers’ recommendations. EC_50_ of the late effect of LEDGINs was determined using virus produced in the presence of a 2-fold dilution series of CX05045, raltegravir or ritonavir. DMSO (vehicle) was included as no-inhibitor control.

To evaluate the kinetics of viral breakthrough, we infected either MT-4 (50,000 cells per well in 1 ml medium in a 24-well plate), stimulated PBMC (500,000 cells per well in 500 μl final volume in a 48 well plate) or 2×10^6^ MDM in 6-well plates with different virus inocula normalized for p24. Virus replication was monitored by quantifying p24 level in the supernatants on successive days using p24 ELISA (Innogenetics).

### HIV-1 entry assay

For the entry assay, 3×10^6^ MT-4 cells were infected with HIV-1_IIIB_ virus produced in the presence of 25 μM of CX05045 or DMSO after normalization for p24 in the presence or absence of 7 μg/ml of DS10000 or 1.5 μM of efavirenz. Cells were incubated with the inhibitors 1 h before infection. Heat inactivated virus was also used as a negative control. Infection was synchronized by incubating cells at 4°C for 1 h and then transferred to 37°C incubator for 2 h. 2 hpi cells were pelleted and treated with trypsin for 60 seconds to remove viruses attached on the surface of cells, and washed three times with PBS. Total RNA extraction, cDNA synthesis and real-time qPCR quantification were performed as described above.

### Time-of-addition

Time-of-addition (TOA) was done in MT-4 cells as described previously [19]. Briefly, 100,000 cells per well in a 96-well plate were infected with HIV-1_IIIB_ at a multiplicity of infection (MOI) of 0.7. Test compounds were used at 50-fold EC_50_ and added every hpi. Cell-free virus released in the supernatant was harvested at 31 hpi. While two-thirds (100 μl) of the harvested supernatants were stored at −80°C to examine the replication capacity of the progeny virion released form the single cycle TOA experiment, the remaining supernatants were used to determine the target blocked by each antivirals in the TOA experiment using p24 ELISA (Innogenetics).

To examine the replication capacity of the viruses released in the TOA experiment, we infected new cells (MT-4 cells, 50,000 per well in a 96-well plate) with normalized inocula for p24 level for those time points where a measurable p24 was detected (e.g., AZT, beyond 4 hpi; raltegravir and CX05045, beyond 8 h and all time points for DMSO-treated viruses). In all cases, we made sure that the estimated concentration of carryover compound to be 50-fold less than the established EC_50_ values of the inhibitors. 4 days postinfection, supernatants were collected and p24 antigen was quantified using p24 ELISA.

### Quantitative PCR analysis of HIV-1 DNA species

MT-4 cells (3.5x10^6^ cells per well in 6-well plates) were infected with HIV-1_IIIB_ produced in the presence of DMSO or 25 μM CX05045 (equivalent of 3.5 μg of p24). The harvested viruses were three times washed with PBS and pelleted as described above. During the qPCR experiment, we added AZT (EC_50_ = 0.02 μM), efavirenz (EC_50_ = 0.0015 μM) or raltegravir (EC_50_ = 0.006 μM) at a concentration of 50- to 100-fold their EC_50_ values as controls for inhibition of RT or integration, respectively. After 2 h of incubation at 37°C, the cells were washed three times with PBS and incubated in fresh medium supplemented with the respective inhibitors. Each time a sample was prepared for qPCR analysis, the supernatant was harvested to monitor the viral replication by p24 ELISA. DNA extractions and quantification of the kinetics of early and late reverse transcripts, 2-long terminal repeat (LTR) circles and integrants were done as described earlier [18,43].

### In vivo PIC nuclear import assay

The PIC nuclear import assay was performed as described before [27]. In brief, 6×10^6^ 293T cells were transfected using PEI with 15 μg of pVpr-IN-eGFP, 15 μg of pD64E (pNL4.3 clone containing the IN_D64E_ inactivating mutation obtained from the AIDS Reference and Reagent Program), and 5 μg of pVSV.G. 6 h posttransfection, the transfection medium was replaced with fresh 0% OptiMEM (supplemented with 50 μg/ml gentamicin) with or without 5-fold EC_50_ of CX05045. Supernatants were collected 48 h post transfection, filtered through a 0.45 μm filter, and then concentrated by ultracentrifugation. Virus inocula equivalent to 250 ng of p24 were used to infect 30,000 HeLaP4 cells/well in 8-chamber slides. 7 hpi, cells were briefly incubated with trypsin (30 sec), fixed with 4% paraformaldehyde and permeabilized with 0.1% Triton-X100 solution in PBS prior to overnight immunostaining of the nuclear lamina with A/C antibody (1/250, Santa Cruz, sc7292). After staining with secondary goat anti-mouse antibody labeled with Alexa Fluor 633 (Molecular Probes) cells were kept in PBS for imaging. Three-dimensional stacks (300 nm per Z-slice) of fixed cells were acquired with a Zeiss LSM 510 laser-scanning confocal microscope using a 63× oil immersion objective. Before quantification, samples were blinded. Multichannel images were contrast stretched (linearly) and assembled and fluorescently labeled PICs (total and nuclear) were quantified using ImageJ software (NIH).

### Single virus FRET assay

Functional fluorescent HIV-1 particles were produced as described above in the in vivo PIC nuclear import assay section with the following modifications: (i) instead of Vpr-IN-eGFP, virions were produced by co-transfecting 293T cells with 3.75 μg of pD64E, 1.25 μg of Vpr-IN-mTFP1 (FRET donor) and 1.25 μg of Vpr-IN-mVenus (FRET acceptor) per well in 6 well plate format, (ii) 6 h post transfection, the transfection mix was removed and replaced with fresh medium supplemented with DMSO or a 5-fold EC_50_ value of either CX05045 or raltegravir, and (iii) viruses were harvested 36 h post transfection, filtered through a 0.45 μm filter and kept at −80°C until use. For the single virus FRET assay, virus preparations were incubated for 3 h at 37°C on a poly-D-lysine (Sigma-Aldrich NV, Bornem, Belgium) coated #1 coverglass (Lab-Tek Chambered Coverglass, VWR International bvba, Leuven, Belgium), washed with PBS (Life Technologies Europe BV, Gent, Belgium) and fixed with 10% formalin (Sigma-Aldrich NV, Bornem, Belgium). Single virus Förster resonance energy transfer (FRET) measurements were carried out on a total internal reflection fluorescence (TIRF) microscope (Olympus IX-71, Olympus NV, Aartselaar, Belgium). The IN-mTFP1 was imaged by objective-type TIRF (PlanApo, 60x, NA 1.45, Olympus) excitation at 150 μW of 445 nm laser light (Cube 445-40c, Coherent, Utrecht, The Netherlands) and wide field detection on an electron multiplying-CCD (ImagEM, Hamamatsu, Louvain-La-Neuve, Belgium) after filtering the mTFP1 emission (HQ485/40 m, Chroma Technology GmbH, Olching, Germany). The IN-mVenus was instantaneously photobleached by 3 mW of 514 nm laser light (Sapphire 514, 100 mW Coherent), after which the IN-mTFP1 was immediately imaged again. FRET was quantified on a single virus basis by super-resolution 2D Gaussian localization of individual virions and extracting the ratio of integrated fluorescence intensity per virion after (F_D_) vs. before (F_DA_) photobleaching: FRET ratio = F_D_/F_DA_. FRET ratios for many (~1-5×10^3^) individual virions were binned in a histogram that was fitted with a normal distribution. Analysis was performed in Igor (WaveMetrics, Inc., Portland, OR, USA). This same protocol was followed for the FRET assay done using LEDGIN-resistant virus, except, instead of the wild type IN, we used Vpr-IN_A128T_-mTFP1 (FRET donor) and Vpr-IN_A128T_-mVenus (FRET acceptor). To test the statistical significance of the data, single virus FRET ratios were used as input for a Student’s *t*-test with unequal variance. A detailed description of this assay will be subject of another publication (Borrenberghs et al., unpublished results).

### Cloning of the Pol bacterial expression construct

The synthetic HIV-1_IIIB_ Pol coding sequence was amplified by PCR from the pcDNA3.1_syn-Gag-Pol construct for which we kindly thank Wagner et al. [44]. The primers (5’-GGGGACAAGTTTGTACAAAAAAGCAGGCTTATTTTTTAGGGAAGATCTGGCCTTC-3’ and 5’-GGGGACCACTTTGTACAAGAAAGCTGGGTATCAGTCCTCGTCCTGCCTGG-3’) contained attB1 and B2 sites allowing the product to be Gateway cloned into pDONR221 (Invitrogen). Next, a D25N substitution was introduced in PR to render it catalytically dead. Site-directed Ligase-Independent Mutagenesis (SLIM) [45] was performed with the following oligos; 5’-CGCCGACGACACCGTGCTG-3’, 5’-CCTGCTGAACACCGGCGCCGACGACACCGTGCTG-3’, 5’-GCCTCCTTCAGCTGGCCACC-3’ and 5’-CCGGTGTTCAGCAGGGCCTCCTTCAGCTGGCCACC-3’ resulting in pDONR221_sPol_PR_D25N_. pDONR221_sPol_PR_D25N_ was recombined with pGGWA and pHMGWA (for which we acknowledge Busso et al. [46]) in an LR Gateway reaction producing pGGWA_sPol_PR_D25N_ and pHMGWA_sPol_PR_D25N_. All constructs were verified by DNA sequencing.

### Purification of recombinant proteins

pGGWA_sPol_PR_D25N_ and pHMGWA_sPol_PR_D25N_ were used to transform competent *E*. *coli* BL21 Star cells (Invitrogen). Briefly, cells were grown to an OD of 0.5, at which point protein production was induced with 0.1 mM Isopropyl β-D-1-thiogalactopyranoside and allowed to continue for 2 h at 25°C. Cells were harvested, lysed and GST-sPol_PR_D25N_ and His-MBP-sPol-PR_D25N_ were affinity purified over Glutathione Sepharose 4 Fast Flow (GE Healthcare) and over HIS-Select Nickel Affinity gel (Sigma) respectively, following the manufacturers’ instructions Purification was monitored via SDS-PAGE and GST-Pol and His-MBP-Pol appeared as single ~140 kDa and ~158 kDa bands, respectively, in the elution fractions after Coomassie staining.

### Pol dimerization assay

For Pol dimerization assays we used the AlphaScreen (PerkinElmer) protein-protein interaction technology is a bead-based technology that allows to study molecular interactions as described before [28]. Briefly, all proteins, compound controls and beads were diluted to their respective working stocks in assay buffer (25 mM Tris/HCl pH 7.5, 150 mM NaCl, 1 mM dithiothreitol, 1 mM MgCl_2_ 0.1% (w/v) BSA, 0.1% (v/v) Tween 20). 5 μl buffer or compound, 5 μl GST-sPol-PR_D25N_ and 5 μl His-MBP-sPol-PR_D25N_ were pipetted in 384-well OptiPlate (PerkinElmer), mixed and incubated at 4°C for overnight. Then we added 10 μl of a mix of glutathione donor and Ni-chelate acceptor AlphaScreen beads (20 μg/ml final concentration each) and the plate was incubated at 23°C for additional 2 h. Eventually the microtiter plate was read in an EnVision Multilabel plate reader (PerkinElmer) and the AlphaScreen signal data were analyzed using Prism 5.0 (GraphPad). Whereas both GST-sPol-PR_D25N_ and His-MBP-sPol-PR_D25N_ were kept constant at 33 nM, the test compounds CX05045, raltegravir or DMSO were titrated in a 1:10 dilution series starting at 100 μM.

### Gel electrophoresis and immunoblot analysis

Protein samples were prepared in 1% SDS. 20 – 30 μg of total protein in each sample was separated by SDS-PAGE (4-12%). Proteins were detected with the respective antibody: rabbit anti-LEDGF/p75 (1:1000 for cell lysate, Bethyl Laboratories. Inc), mouse monoclonal anti-HIV-1 IN (IN2, 1:10,000 for viral lysates and 1:2000 for cell lysates, Abcam), mouse anti-HIV-1 CA (1:10,000, AIDS reagents Program). Visualization was performed using chemiluminescence (ECL+, Amersham Biosciences, Uppsala, Sweden).

### Electron microscopy

HUT78_IIIB_ cells were counted and washed twice with PBS and grown in the presence of DMSO or 25-fold EC_50_ of inhibitor (raltegravir, CX05045 or ritonavir) for 24 to 36 h. Subsequently, cells were washed twice with PBS and incubated with fresh medium with or without the indicated compounds. After 6 days cells were harvested, pelleted, and fixed with 2.5% glutaraldehyde overnight at 4°C. Cell pellets were post-fixed with OsO4 (1% in ddH2O; Plano, Wetzlar, Germany), block-stained with uranyl acetate (2% in ddH2O; Merck, Darmstadt, Germany), dehydrated stepwise in graded alcohol, immersed in propylenoxide and embedded in Epon (Serva, Heidelberg) with polymerisation at 60°C for 48 h. Ultrathin sections (60–80 nm) were cut using an ultramicrotome (Ultracut S or UCT; Leica, Germany) and stained with 2% uranyl acetate and lead citrate. Transmission electron microscopy was performed with an EM 902 (Zeiss) operated at 80 kV and the images were digitised using a slow-scan charge-coupled-device camera (Pro Scan; Scheuring, Germany).

### Statistical analysis

Statistical analysis was performed using GraphPad Prism version 5.0 (http://www.graphpad.com/prism). All data points were included in the analysis for significance and paired comparisons were carried out using Student’s *t* test, 1-way or 2-way ANOVA. The cumulative relative frequency distribution of the eGFP labeled PICs was analyzed using the two-tailed Kolmogorov-Smirnov test using XLSTAT 2013 for windows (http://www.xlstat.com).

## Competing interests

Authors declare no conflict of interests.

## Authors’ contributions

BAD and ZD conceived and designed the experiments and wrote the manuscript. BAD, RS, JD, CW, WT, SV and BVR performed biochemical and virological experiments and analyzed data. DB, JH and JH designed and performed the single molecule FRET assay and analyzed data. NB performed the electron microscopy experiment and revised the manuscript. JDR, RG and FC analyzed data and revised the manuscript. ZD supervised the project. All authors read and approved the final manuscript.

## Supplementary Material

Additional file 1: Figure S1 HIV-1 produced in the presence of CX05045 failed to replicate. Figure S2. LEDGINs do not affect virus production or gRNA packaging. Figure S3. Nuclear import of HIV-1 PICs produced in the presence of CX05045. Table S1. Estimates of the average delay of TOA of compounds and 95% CI for 50% 46 inhibition of single cycle HIV-1 replication and infectivity in MT-4 cells. Click here for file
