# Correction to: LEDGINs inhibit late stage HIV-1 replication by modulating integrase multimerization in the virions

**DOI:** 10.1186/s12977-020-00530-4

**Published:** 2020-07-29

**Authors:** Belete Ayele Desimmie, Rik Schrijvers, Jonas Demeulemeester, Doortje Borrenberghs, Caroline Weydert, Wannes Thys, Sofie Vets, Barbara Van Remoortel, Johan Hofkens, Jan De Rijck, Jelle Hendrix, Norbert Bannert, Rik Gijsbers, Frauke Christ, Zeger Debyser

**Affiliations:** 1 Department of Pharmaceutical and Pharmacological Sciences, Laboratory for Molecular Virology and Gene Therapy, KU Leuven, Kapucijnenvoer 33, 3000 Louvain, Flanders Belgium; 2 Laboratory for Photochemistry and Spectroscopy, KU Leuven, Celestijnenlaan 200F, 3001 Heverlee, Flanders Belgium; 3 Centre for HIV and Retrovirology, Robert Koch Institute, Nordufer 20, 13353 Berlin, Germany

## Correction to: Retrovirology (2013) 10:57 10.1186/1742-4690-10-57

Following publication of their article [1], the authors realized that an error occurred in Figure 3f during mounting of the Figure for the p55 band (2 different blots were used, which was not indicated in the figure legend). Therefore, the authors would like to replace Fig. 3f with a new one based on one single experiment and blot. This new Figure does not affect any results or conclusions.

**Fig. 3:**
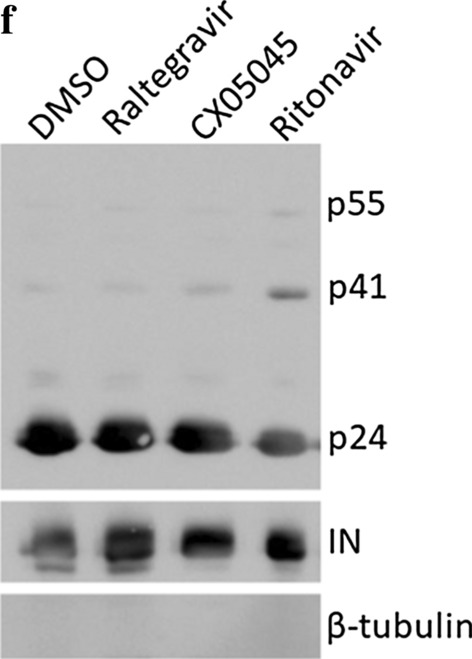
**f** Western blot analysis of viral proteins in HuT78_IIIB_ producer cells or the corresponding cell-free viruses produced in the presence of the indicated compounds. β-tubulin was used as loading control. Scale bars represent 500 nm
