# Supplementary material for: LEDGINs inhibit late stage HIV-1 replication by modulating integrase multimerization in the virions
Source: Retrovirology. 2013 May 30;10:57. doi: 10.1186/1742-4690-10-57 (PMC3671127; doi:10.1186/1742-4690-10-57)
Supplement: Additional file 1: Figure S1 — HIV-1 produced in the presence of CX05045 failed to replicate. Figure S2. LEDGINs do not affect virus production or gRNA packaging. Figure S3. Nuclear import of HIV-1 PICs produced in the presence of CX05045. Table S1. Estimates of the average delay of TOA of compounds and 95% CI for 50% 46 inhibition of single cycle HIV-1 replication and infectivity in MT-4 cells. [file 1742-4690-10-57-S1.pdf]

Supporting Information

LEDGINs inhibit the post-integration stage of the HIV-1 replication by modulating integrase multimerization in the virions

Belete Ayele Desimmie[1], Rik Schrijvers[1], Jonas Demeulemeester[1], Doortje Borrenberghs[2], Caroline Weydert[1], Wannes Thys[1], Sofie Vets[1], Barbara Van Remoortel[1], Johan Hofkens[2], Jan De Rijck[1], Jelle Hendrix[2], Norbert Bannert[3], Rik Gijsbers[1], Frauke Christ[1], Zeger Debyser[1]*

Legends of Supporting Figures

Figure S1. HIV-1 produced in the presence of CX05045 failed to replicate. HIV-1 replication monitored in MT-4 cells (infected for 1 h and washed 3 times to remove as much carryover free compounds as possible) by p24 quantification for (**A**) NL4.3 and (**B**) HXB2D produced in the presence of DMSO, raltegravir, CX05045 or ritonavir in 293T cells. While ritonavir delayed breakthrough for a few days compared to DMSO and raltegravir, CX05045 apparently crippled the progeny virions and the p24 level rather goes down after splitting. Although cells were split on day 6 and 9 post infection to prevent overgrowth and cell death p24 sampling continued, all but CX05045-pretreated virus replicates. (**C, D**) Analysis of MOI dependency of the infectivity of (**C**) NL4.3.and (**D**) YU-2 viruses, we evaluated viral breakthrough with 5, 50 or 500 ng p24 equivalent inoculum in MT-4 cells and MDM, respectively. Virus produced in the presence of

CX05045 again did not replicate compared to the DMSO-, raltegravir- or ritonavir-pretreated viruses during the course of the experiment.

Figure S2. LEDGINs do not affect virus production or gRNA packaging

HIV-1 (NL4.3, HXB2D and $NL4.3_{A128T}$) were produced in the presence of CX05045 (5 µM), raltegravir (0.03 µM) or ritonavir (0.3µM). (**A**) Virus production and (**B**) gRNA packaging as quantified by p24 ELISA and RT-qPCR for each strains. Mean values ± standard deviations are shown. ***p < 0.0001; 2-way ANOVA.

Figure S3. Nuclear import of HIV-1 PICs produced in the presence of CX05045. Representative images of HeLaP4 cells infected with HIV_{DMSO} and $HIV_{CX05045}$. 7 hpi with HIV-IN-eGFP (*green*), cells were fixed and immunostained with the nuclear lamina A/C primary antibody and Alexa Fluor 633 labeled secondary antibody (*blue*). Three-dimensional confocal z stacks were acquired with maximum projection and to acquire the images shown here only five stacks were overlaid centered in the middle of the nucleus. Cell shapes are outlined in white. White arrows indicate nuclear PICs.

Supporting Tables

Table S1. Estimates of the average delay of TOA of compounds and 95% CI for 50% inhibition of single cycle HIV-1 replication and infectivity in MT-4 cells.

Delay time estimates (h)[a]

	**Parameter**	**AZT**	**Raltegravir**	**CX05045**	**Ritonavir**
Single cycle replication inhibition[b]	Time delay ($t_{1/2}$)	7.0	12.4	12.1	25.7
	95% CI for delay	6.6 – 7.4	12.2 – 12.5	11.7 – 12.5	25.5 – 26.0
	Goodness fit (R^2)	0.9112	0.9933	0.9167	0.7658
Infectivity inhibition[c]	Time delay ($t_{1/2}$)	4.0	11.3	24.5	20.6
	95% CI for delay	3.7 - 4.2	11.1 - 11.5	24.3 – 24.8	19.5-21.7
	Goodness fit (R^2)	0.9612	0.9862	0.8668	0.7203

[a] Estimates of the time delays ($t_{1/2}$; h) yielding 50% inhibition of HIV replication. [b] TOA was performed to determine the estimated time delays for each compounds to corroborate with the established target for each class of inhibitors. [c] The level of p24 protein in the supernatants of MT-4 cells incubated with supernatants harvested from each time points of the TOA experiment for each compound and relative replication capacity of the viruses was determined. The calculation assumes the same replication rate across all compounds and all time points.

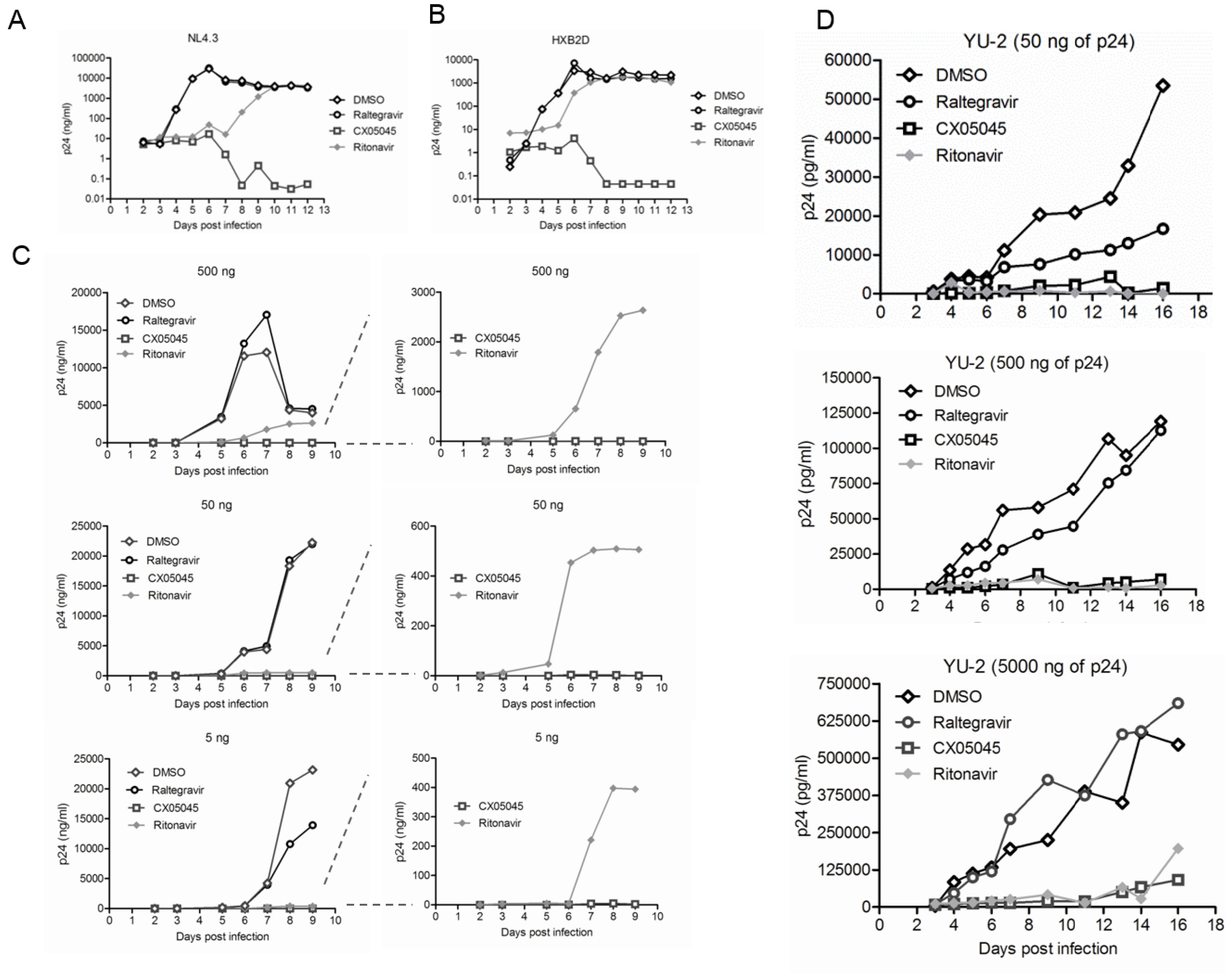
A
NL4.3
p24 (ng/ml)
Days post infection
DMSO
Raltegravir
CX05045
Ritonavir
B
HXB2D
C
500 ng
50 ng
5 ng
D
YU-2 (50 ng of p24)
YU-2 (500 ng of p24)
YU-2 (5000 ng of p24)
p24 (pg/ml)

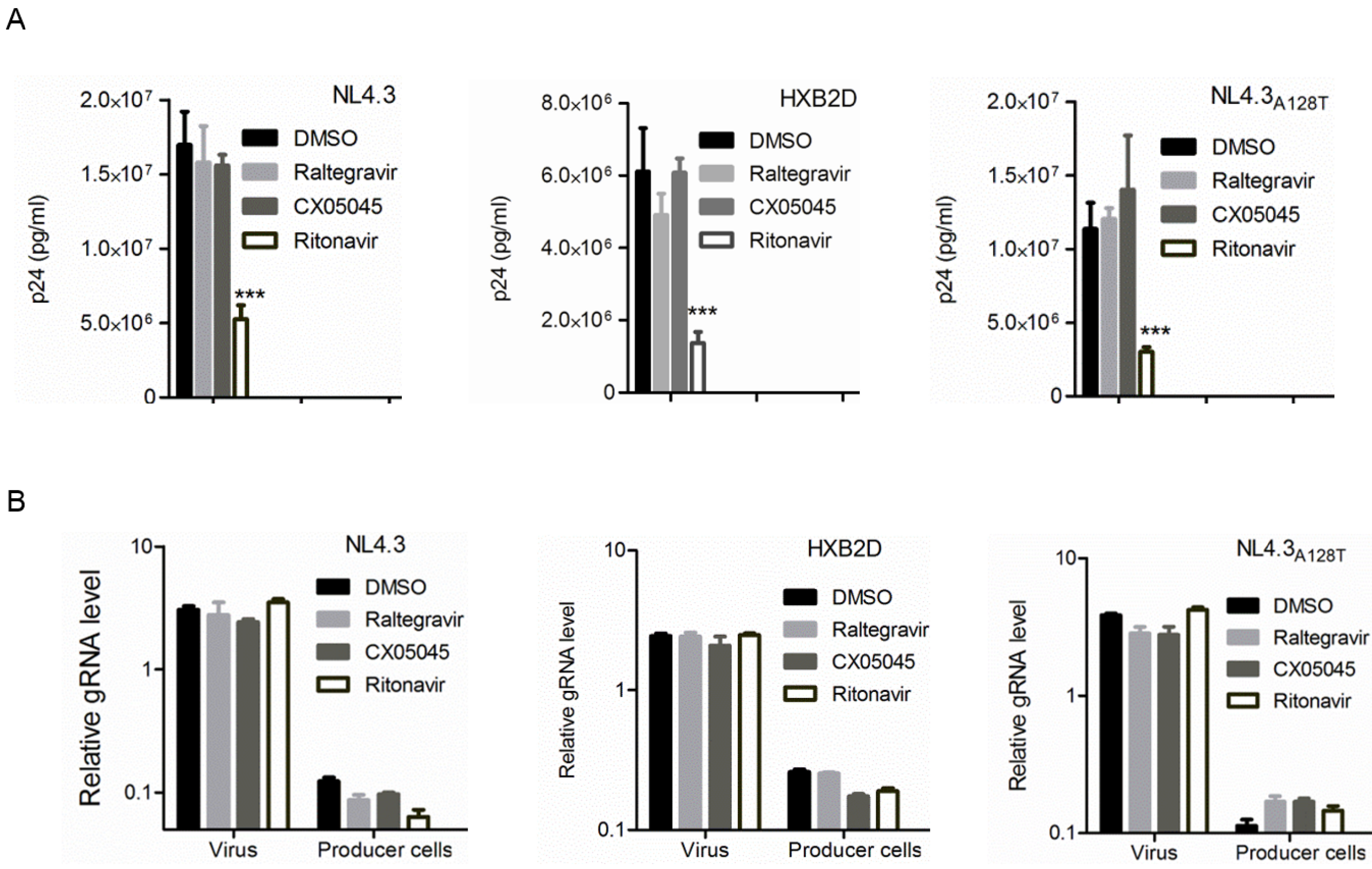
A
NL4.3
HXB2D
NL4.3A128T
p24 (pg/ml)
2.0x10^7
1.5x10^7
1.0x10^7
5.0x10^6
8.0x10^6
6.0x10^6
4.0x10^6
2.0x10^6
0
DMSO
Raltegravir
CX05045
Ritonavir
***
B
Relative gRNA level
10
1
0.1
Virus
Producer cells

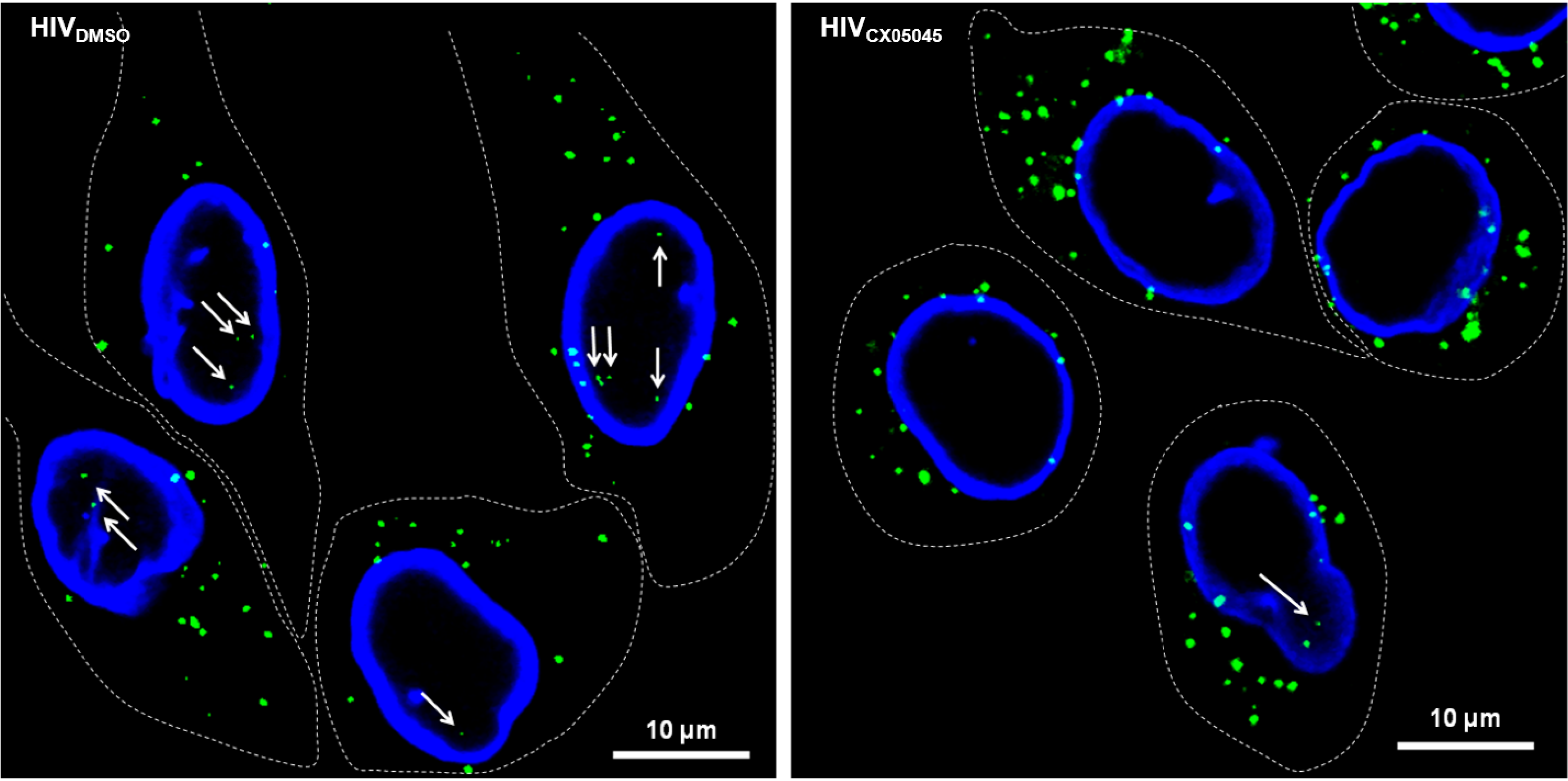

HIV$_{DMSO}$
10 µm
HIV$_{CX05045}$
10 µm
